# One Patient, Two Problems: Applying Short Bowel Syndrome Management Principles in Inflammatory Bowel Disease patients

**DOI:** 10.1007/s11894-026-01054-1

**Published:** 2026-08-25

**Authors:** Adeeti J. Chiplunker, James Burton, Bryn Koehler, Matthew L. Bechtold

**Affiliations:** 1 Division of Gastroenterology, Hepatology and Nutrition, Ohio State University Wexner Medical Center, 395 W 12th Avenue, FOT - Floor 2, Columbus, OH 43210 USA; 2 Department of Gastroenterology, Hepatology, and Nutrition, University of Louisville, Louisville, KY USA; 3 Division of Gastroenterology and Hepatology, University of Missouri, Columbia, MO USA

**Keywords:** Inflammatory bowel disease, Short bowel syndrome, Intestinal failure, High ileostomy output

## Abstract

**Purpose of Review:**

Surgery for inflammatory bowel disease (IBD) often results in alterations in gut function beyond the anatomical changes. While some patients develop short bowel syndrome (SBS), a significant proportion of IBD patients benefit from pharmacologic and dietary interventions for SBS even in the absence of true SBS or IF.

**Recent Findings:**

While no formal criteria exist to clearly define the presence of SBS in IBD patients, the presence of specific features in the surgical and clinical history are crucial to the rapid identification of SBS or IF in IBD patients. Anatomy specific dietary considerations and interventions provide significant benefits and lay the foundation for a structured approach to optimizing anti-motility and adjunctive therapies.

**Summary:**

IBD patients with post-surgical anatomy benefit from management strategies used in patients in SBS. Optimal dosing of anti-motility agents and oral rehydration solution form the foundations of management in all practice settings.

## Introduction

Inflammatory bowel disease, specifically Crohn’s disease, has been well described as a risk factor for intestinal failure (IF) and short bowel syndrome (SBS) [1]. Intestinal failure occurs when the gut is unable to absorb the minimum necessary macronutrients, water, and/or electrolytes can occur required to maintain health and a concurrent necessity for intravenous supplementation of these components [2]. SBS is generally defined as having < 200 cm of small bowel remaining when measured from the ligament of Treitz but IF may or may not be present depending on the residual bowel anatomy [3]. Patients with SBS are more likely to have intestinal failure if there is < 100 cm of small bowel remaining in ostomy patients or if there is < 65 cm of small bowel remaining in patients with at least 50% of the residual colon in continuity [3]. Approximately 22% of SBS cases are due to Crohn’s disease [4]. However, it is important to note that patients with IBD may meet criteria for IF in the absence of SBS. The full extent of IBD patients who meet criteria for IF in the absence of SBS is not known although rough estimates vary from 8 to 30% [5].

Despite the considerable overlap between IBD and SBS, these two conditions have historically been managed separately by two teams. The IBD provider is responsible for the medical management of their IBD and the SBS/Nutrition support team responsible for parenteral support and titration of anti-diarrheal agents. However, with this split model, appropriate communication may be delayed or missed, particularly if easy access to medical records from each party is not available. As such, improving recognition of IF in IBD patients as well as providing easily applicable principles of symptom management is the goal of this review.

## Defining IF and SBS in IBD

Before considering a diagnosis of intestinal failure or short bowel syndrome in an IBD patient, it is critical to first assess their disease activity status. Active bowel inflammation significantly impairs residual bowel function and will confound the picture when trying to accurately identify IF. Disease activity assessment should include objective measurements including fecal calprotectin, cross-sectional imaging with enterography or intestinal ultrasound, and endoscopy as applicable. If active disease is present, further optimization of their disease control is paramount with follow up measurement of objective testing to confirm remission as part of a treat-to-target approach such as is outlined in the STRIDE-II consensus recommendations [6].

After excluding active disease, the next step in evaluating a patient with IBD in whom a diagnosis of IF or SBS is being considered is to identify the remaining small bowel length and the residual anatomy. Reviewing prior operative notes if available is a must. Ideally, the most recent operative report will contain a measurement of the residual small bowel but this is often not the case. Gross pathology reports can be helpful if operative reports are not available or the residual bowel was not measured as these reports will include the length of the removed specimen. If an exact number is not available or cannot be extrapolated from gross pathology reports, a history of 3 or more bowel resections should prompt further investigation into the presence of IF.

Even in patients who do not meet length criteria, the presence of the following clinical features should raise the index of suspicion for IF: Ostomy output > 1.5 L/day, recurrent low sodium or magnesium, low body mass index in combination with elevated blood urea nitrogen, urine sodium < 20mmol/L, chronic diarrhea despite mucosal healing, frequent hospitalization for dehydration, and unexplained weight loss. While these do not define IF, the presence of these clinical features are indicators that bowel function is compromised and parenteral hydration or electrolyte support may be required.

### Anatomical Considerations

There are three key anatomical considerations when managing IF in IBD patients. Ileocecectomy is the most common surgery performed in patients with Crohn’s disease [7]. Even with advent and widespread use of biologic therapies, rates of surgical resection remain high, ranging from 16% at 1 year from diagnosis to as high as 47% at 5 years from diagnosis [8]. Similarly, approximately 35% of patients will require a subsequent resection by 10 years [8]. As such, patients with distal ileal disease/ileocecal involvement are at risk of losing a substantial portion of the ileum, which has important consequences for gut motility. The ileum serves as a “brake” for the system by producing peptide YY, glucagon-like peptide-1 (GLP-1) and glucagon-like peptide-2 (GLP-2) in response to nutrient stimulation which results in slowing of gastric and intestinal motility [4]. The ileum is the primary site of bile acid reabsorption so patients who have undergone ileocecectomy or distal ileum resection are at risk for bile acid malabsorption related diarrhea in circumstances where the colon is continuity with the remnant small bowel.

Patients with ulcerative colitis may undergo total colectomy with or without ileo-anal pouch anastomosis (IPAA) creation for medically refractory disease. The colon plays a critical role in driving intestinal adaptation to small intestinal resection, namely in the production of glucagon-like peptide-2 from enteroendocrine L cells present in the distal small bowel and colon [9]. The colon also plays a key role in water absorption in the native anatomic state, absorbing 90% of the 1.5–2 L of fluid entering from the ileum and absorbing as much as 4–5 L of fluid if needed [4]. As such, continuity of the residual small bowel with the colon is a predictor of successful parenteral nutrition weaning in patients with short bowel syndrome [10]. In the absence of a colon, patients with an end-ileostomy or IPAA may struggle with high fluid losses and/or an inability to rapidly replenish fluid and electrolytes after periods of increased fluid loss such as acute diarrheal illness, fever, or periods of heavy sweating during intense exercise.

About 5% of patients with Crohn’s disease have upper GI involvement [11]. Those with a stricturing phenotype with symptomatic duodenal stenoses may have undergone gastrojejunal bypass surgery with concurrent jejunojejunal bypass to circumvent the stenotic area, particularly if the operation occurred before stricturoplasty was more widely utilized. These patients may subsequently struggle with malabsorption not dissimilar to that experienced by patients who have undergone Roux-en-Y gastric bypass surgery for obesity. In particular, the reduction in cholecystokinin (CCK) signaling significantly impacts the necessary slowing of gastric motility and appropriate release of bile and pancreatic enzymes required for optimal digestion and absorption of nutrients in the proximal small bowel [4].

## Laying the Foundations of Symptom Control

### Use of Anti-motility Agents

**Fig. 1:**
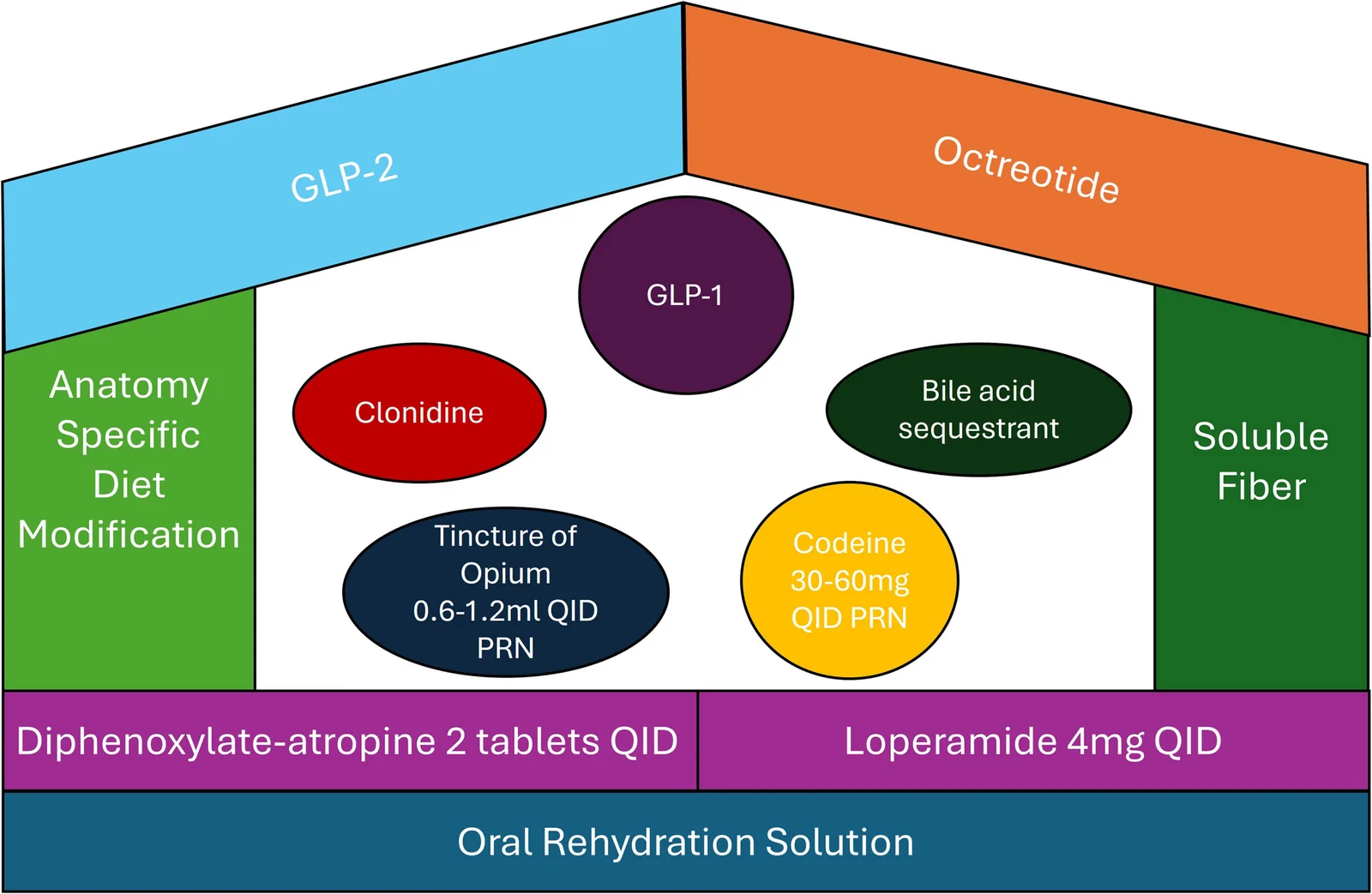
Structured approach to medical and dietary management of IBD patients with and without IF

One of the primary goals in managing a patient with IF is controlling the quantity and frequency of stool output to reduce overall fluid losses. Optimal dosing of anti-motility agents, specifically with the opioid-antagonist agents loperamide and diphenoxylate-atropine plays a critical role in managing stool output. The recommended dose of loperamide recommended is 16 mg/day, which breaks down to 4 mg (two tablets) taken four times daily (Fig. 1.). While doses as high as 32 mg/day have been used in clinical practice, nausea may limit achieving this dose and there may be minimal symptom benefit with doses higher than 16 mg/day, particularly when used as monotherapy. Serious side-effects such as increased QTc interval or life-threatening ventricular arrythmia have been reported at loperamide total daily doses greater than 60 mg/day [12].

Using loperamide in combination with diphenoxylate-atropine is a common strategy in managing stool output in SBS patients. The maximal recommended dose of diphenoxylate-atropine is two tablets taken four times daily in combination with loperamide (Fig. 1.). As both diphenoxylate and atropine can cross the blood-brain barrier, doses in excess can cause over-sedation, dizziness, and dry mouth due to the anti-cholinergic effects of atropine.

The post-prandial period is often the most symptomatic for most patients whether due to loss of physiologic controls or due to the osmotic effects of the food intake itself. As such, the optimal timing for administration is 30–60 min prior to breakfast, lunch, and dinner and the fourth dose at bedtime on a scheduled basis rather than as needed in response to diarrhea. This timing is important as it promotes global slowing of the gut in preparation for the stimulus of a meal and increases the duration that the intestinal contents spend in contact with the small bowel, thereby increasing the absorption of nutrients. Even if that specific meal is not eaten, scheduling the anti-motility agent at these common time points during the day ensures a consistent level of dosing throughout the day as a proactive rather than reactive approach.

Tincture of opium (anhydrous morphine 10 mg/mL) can be a useful adjunct should high stool output persist after optimal dosing of loperamide, and diphenoxylate-atropine has been implemented (Fig. 1.). Suggested dosing is 0.6 to 1.2mL every 6 h as needed as higher doses increase the risk of sedating side effects such as dizziness and drowsiness when used in combination with loperamide and diphenoxylate-atropine [13]. Tincture of opium has the benefit of being in liquid form which can aid in more rapid absorption. Unfortunately, this product can have an unpleasant taste and is not always available at commercial pharmacies, which can limit its use.

Codeine is a reasonable alternative to tincture of opium as an adjunct to loperamide and diphenoxylate-atropine. Suggested dosing is 30-60 mg every 6 h as needed to reduce the risk of sedating side effects (Fig. 1.). It has the benefit of an acceptable taste profile in tablet form and better pharmacy availability although insurance coverage varies, and prior authorization is sometimes needed, which can limit its use.

A structured anti-motility agent regimen can be implemented in IBD patients with and without IF. A stricturing Crohn’s phenotype is not an absolute contraindication for anti-motility agent use. A reasonable approach is to start by maximizing loperamide prior to adding diphenoxylate-atropine and doing so in a stepwise fashion. A similar approach can be applied in patients with symptomatic obstructive episodes due to abdominal adhesions. While there is no specific recommended algorithm for titrating anti-motility agent therapy in these patients, doing so with close monitoring and frequent patient feedback are keys to success.

### Oral Fluid Intake

A critical but often overlooked component of managing stool output is the osmolality of oral fluid intake. Hypo-osmolar fluids like water and hyper-osmolar fluids like juice and soft drinks can significantly contribute to diarrhea. As passive water absorption alone in these patients is insufficient to maintain hydration status, an emphasis on oral fluids that mimic isotonic composition is critical.

Oral rehydration solutions (ORS) formulated to the World Health Organization’s (WHO) standard osmolality of 245mmol/kg are the mainstay of management in acute infectious diarrheal illness but can also be utilized in chronic conditions such as SBS and IBD [14]. ORS contain sodium and glucose in order to leverage the function of the sodium-glucose cotransporter system and facilitate active water absorption, a process which remains intact even in diseased states of the colon or the absence of the colon altogether [15].

ORS has been shown to improve biochemical indicators of hydration status and kidney function, reduce ostomy output, reduce hospital readmissions for dehydration and AKI in patients with high ileostomy output [16]. And yet, despite the increased visibility and availability of ORS products, outside of specialized centers experienced in the care of IF patients, few patients, if any, receive appropriate counseling on the type of oral fluid intake recommended. Patient education plays a significant role in improving adherence to recommended ORS intake and can be readily implemented in both the inpatient and outpatient setting [17, 18].

Several commercially available ORS products are available though not all of these meet the osmolality criteria advocated by the WHO or fall within the range of maximal fluid absorption of 200-260mmol/kg [19]. Most of the commonly utilized commercially available pre-mixed electrolyte beverages and sports drinks have an osmolality greater than 300mmol/kg whereas the powdered formulations tend to fall within the optimal range [14, 19]. Additional home-made ORS recipes have also been formulated to increase the variety and accommodate taste preferences and are easily searchable on the internet [20].

The addition of ORS plays a significant role in managing hydration in IBD patients. IBD patients who have undergone colectomy with either end ileostomy or IPAA or those with diverting loop ileostomy can benefit from the addition of ORS. Even those for whom ostomy output is at goal of < 1.5 L per day or who have no issues with dehydration can benefit from ORS during periods of increased fluid losses such as heavy exercise, periods of increased sweating during hot weather, self-limited gastroenteritis episodes or during febrile illness. The absence of the colon limits the ability to compensate with increased fluid absorption to counteract dehydration in these settings and as such, ORS can help speed recovery and potentially minimize the need for hospitalization for dehydration [16]. Similarly, while IBD patients with an intact colon are often able to maintain their hydration status with hypotonic oral fluids alone, patients who have lost 50% or more of their colon or those with a fully intact colon but active colitis do not have the ability to adapt [21]. These patients also benefit from adding ORS to their oral fluid intake to sustain normal hydration status even if they otherwise do not meet criteria for SBS or IF.

### Diet Modification

Dietary recommendations in patients with SBS are dependent on the baseline anatomy, specifically the presence or absence of the colon in continuity. SBS patients with their colon in continuity should aim to intake 50–60% of their calories from complex carbohydrates with a particular focus on soluble fiber [22–24]. Soluble fiber helps modulate gut dysbiosis by promoting the growth of the commensal microbiome, solidifies stool and increases colonic transit time, all of which can help in the management of diarrhea [22, 24]. Soluble fiber also undergoes bacterial fermentation in the colon to form short chain fatty acids (SCFAs) which can be salvaged as additional calories by the colon [25]. Approximately 20–30% of calories should be from fat with a specific focus on essential fatty acids and medium chain triglycerides (MCTs) to enhance absorption [23, 25]. This can reduce steatorrhea but is important to note that fat restriction may be insufficient to reduce diarrhea in patients with severe SBS and these patients may be dependent on dietary fats to sustain the density of caloric intake. The remainder of caloric intake (20%) should be from proteins, which are effectively absorbed by patients with SBS (Table 1) [22, 24].

**Table 1:** Anatomy Based Diet Composition Recommendations

	Colon in Continuity	Colon Absent
Carbohydrate	50–60% of calories Limit simple sugars Complex carbohydrates	40–50% of calories Limit simple sugars Complex carbohydrates
Fiber	Soluble Fiber	Soluble fiber (texture modified)
Fat	20–30% of calories Short and medium chain triglycerides	30–40% of calories Long chain triglycerides Limit medium chain
Protein	20% of calories	20% of calories

Dietary recommendations for SBS patients with small bowel ostomy anatomy differ from those with colon in continuity. These patients should derive 40–50% of calories from complex carbohydrates. This is lower than that recommended for those with colon in continuity as the SCFA salvage activity has been lost and simple carbohydrates are more likely to increase ostomy output due to their osmotic effects. Soluble fiber still plays a valuable role in these patients by increasing the viscosity of the stool which can have important positive effects on quality life and enterostomal appliance issues [26]. Marshmallow intake has also been shown to have a positive effect on frequency of ostomy appliance changes and decreasing the volume of ileostomy output due to the thickening properties of gelatin, though moderation should be used to avoid excessive intake of simple carbohydrates [27]. Patients with small bowel ostomy anatomy can tolerate a higher fat intake than those with colon in continuity. As such, fats can comprise 30–40% of the diet but MCT supplementation is not necessary as these can decrease carbohydrate and protein absorption in this population [25]. Protein recommendations are the same as those for patients with colon in continuity (Table 1) [22, 24].

The diet recommendations for patients with SBS are readily applied to IBD patients with similar anatomy. Soluble fiber plays a vital role in IBD beyond its ability to thicken stool output. Soluble fiber has been shown to positively modulate the gut dysbiosis seen in IBD while the SCFAs produced during the fermentation process interact with Toll-like receptors and generate an anti-inflammatory response [28]. In ileostomy patients, soluble fiber is still able to modulate the gut microbiome even though colonic SCFA production has been lost [29]. To encourage adequate fiber intake, texture modification may be necessary in patients with stricturing disease or ostomy anatomy to reduce the risk of obstructive symptoms [29]. For example, whole fruits may not be tolerated but peeled fruits in smoothie or canned form may be safely consumed. Vegetables may be more easily consumed when peeled, cooked and blended into soup form when even soft-cooked textures are not tolerated. This dynamic process is best explored with the assistance of a dietitian, but if this resource is not available, the Crohn’s and Colitis Foundation provides excellent guidance with recipes designed for patients with stricturing disease [30].

While soluble fiber can benefit IPAA patients with respect to stool viscosity and output and reduction in fecal incontinence, the fermentation of anti-inflammatory SCFAs does not produce consistent effect [29]. Intake of fruits and vegetables has been shown to increase microbial diversity of the pouch and reduce the risk of pouchitis, although texture modification may be required in some patients [31].

## Adjunctive Therapies

Once a solid foundation of scheduled anti-motility agents, ORS intake and diet modification has been initiated, additional agents may be utilized if stool output remains high.

### Bile Acid Sequestrants

Bile acid reabsorption occurs in the terminal ileum and results in reabsorption of approximately 95% of bile acids for enterohepatic recirculation back to the liver [32]. In patients with bile acid malabsorption and < 100 cm of terminal ileum removed (e.g.: post-cholecystectomy, active ileitis in Crohn’s disease), bile acid sequestrants such as cholestyramine or colestipol can provide meaningful symptom relief. Bile acid diarrhea is primarily a secretory diarrhea driven by the colonic mucosa in response to exposure to unabsorbed bile acids [32]. As such, patients without a colon are unlikely to have a clinically meaningful response to bile acid sequestrant therapy. In SBS patients who have lost > 100 cm of ileum, use of bile acid sequestrants worsens steatorrhea due to further loss of the already depleted bile acid pool. Caution with bile acid sequestrant therapy is advised in patients for whom their remaining ileal length is unknown or those who are borderline for 100 cm of ileum remaining. Any trial of bile acid sequestrant therapy in these patients requires close monitoring for worsening of stool output.

### Clonidine

Clonidine is an α_2_-agonist that has been shown to have anti-diarrheal efficacy due to its antimotility properties and prolongation of intestinal transit time [23, 33]. In clinical practice, clonidine has shown efficacy for reducing ostomy output in several small case series, demonstrating a clinically significant reduction in fecal output and reduction of fecal sodium losses (Fig. 1.) [34, 35]. Transdermal applications can improve compliance and reduce the risk of rebound hypertension, but use may be contraindicated in patients with low blood pressure.

### Octreotide

Octreotide is a somatostatin analog with slowing effects on intestinal transit time, reduction in intestinal secretion, and promotion of intestinal electrolyte absorption [36]. Octreotide is already being utilized in the management of chemotherapy associated diarrhea and diarrhea secondary to carcinoid and vasoactive intestinal peptide secreting tumors [36]. Octreotide has shown some efficacy in reducing ostomy output in patients with SBS and can be used in patients for whom optimal dosing of anti-motility agents has been insufficient and alternative therapies are not available or contraindicated [23].

### GLP-1

Exogenous GLP-1 receptor agonist administration can cause a wide variety of gastrointestinal side effects, specifically resulting from reductions in GI motility and prolongation of intestinal transit time [37]. These effects on GI tract motility naturally led to consideration of their applicability in patients with SBS. GLP-1 receptor agonists have shown efficacy in reducing parenteral support volume and reducing stool output in small observational studies [38]. A deeper understanding of metabolic alterations and the rising incidence of obesity in IBD patients has led to further studies evaluating the application of GLP-1 receptor agonists in the treatment of IBD. GLP-1 receptor agonists show potential benefits in reducing hospitalization, lowering inflammatory biomarker values, and promoting effective weight loss in obese IBD patients while maintaining tolerability profile like that of patients without IBD [39]. While further studies are needed in this area and insurance coverage challenges remain, GLP-1 receptor agonists can be successfully utilized in IBD and SBS patients to manage high stool output but have limited effect on increasing small bowel absorption.

### GLP-2

GLP-2 secretion in the distal ileum and colon increases in response to intestinal resection and plays a critical role in the bowel adaptation process that occurs after resection [40]. GLP-2 stimulates crypt cell proliferation in the epithelia of the stomach, small bowel, and colon, thereby enhancing the absorptive capacity of the bowel [41]. Understanding these important effects led to the development of GLP-2 receptor agonists to augment the bowel adaptation process. In clinical trials, the GLP-2 receptor agonist teduglutide resulted in clinically significant reductions in parenteral support volume of ≥ 20% and a reduction in the number of days of parenteral support [42, 43]. Real world studies after FDA approval of teduglutide showed a 40% reduction in PN requirements and complete discontinuation of PN in 20–30% of patients [38]. Additional real-world experience also showed significant improvements in physical and mental quality of life components associated with reduction in number of PN days, and a reduction in liver enzymes [38, 44]. As with other adjunctive therapies, GLP-2 therapy is most effective in patients who have already implemented anti-motility, oral fluid, and anatomy specific diet recommendations. It is important to note, however, that teduglutide therapy is costly, with an average estimated annual cost of $300,000 per patient in the US and consideration of the benefit and clinical meaningfulness of treatment is warranted before starting therapy [45].

Due to the trophic effects of GLP-2 receptor agonist administration on the intestinal mucosa, there is a potential risk for bowel obstruction symptoms, which is of particular concern in IBD patients with stricturing disease. In a small case series of 13 patients with Crohn’s disease who were treated with teduglutide, only one patient experienced obstructive episodes which subsequently resolved when teduglutide dosing was reduced to every other day [46]. In a similar small case series of 9 patients with Crohn’s disease who were treated with teduglutide, 2 patients experienced obstructive symptoms with 1 of those requiring hospitalization and discontinuation of therapy while the other had resolution with every other day dosing [47]. Despite the small size of these case series, a reduction in parenteral support volume was achieved in almost all patients, even in those on every other day dosing, highlighting the efficacy and safety of teduglutide use in IBD patients with SBS [46, 47].

## Conclusions

Early recognition of features of SBS or IF in an IBD patient is paramount for minimizing complications while also improving quality of life and preventing cycling through biologic medications for lack of response. Even patients with ileo-anal pouch anastomosis or ileostomy anatomy may benefit from a structured management approach utilized in patients with SBS.

Optimal management of the IBD patient with symptoms of IF or SBS begins with building a solid foundation rooted in optimal anti-motility agent dosing and an emphasis on intake of oral rehydration solutions. Diet adjustment based on the residual anatomy is also key to reducing symptom burden while potentially gaining additional anti-inflammatory benefits as in the case of soluble fiber in patients with an intact colon. Adjunctive therapies such as octreotide and teduglutide can provide additional benefit in expert hands but the foundation of anti-motility agent dosing and ORS intake can provide rapid symptom improvement. This practical approach rooted in expert clinical practice provides a management framework that is applicable in all practice settings.

## Key References

- Carol Rees Parrish MS R. Staying Hydrated with Oral Rehydration Solution 2021. https://www.shortbowelsyndrome.com/Content/pdf/Recipe_Book_DIGITAL.pdf
  - ○ Contains practical recipes for oral rehydration solution options to accommodate various taste and texture preferences.
- Gold S, Park S, Katz J, et al. The Evolving Guidelines on Fiber Intake for Patients with Inflammatory Bowel Disease; From Exclusion to Texture Modification. Curr Gastroenterol Rep. 2025;27:23.
  - ○ Review of different fiber types including fermentable and soluble fiber, outlines options for texture modification depending on IBD disease activity status
- Crohn’s and Colitis Foundation. Gut Friendly Recipes. 2026. https://www.crohnscolitisfoundation.org/patientandcaregivers/gutfriendlyrecipes.
  - ○ Practical recipes for IBD patients including options for patients with ostomy or strictures.

## Data Availability

No datasets were generated or analysed during the current study.
